# A population-based follow-up study shows high psychosis risk in women with PCOS

**DOI:** 10.1007/s00737-021-01195-4

**Published:** 2021-11-29

**Authors:** Salla Karjula, Riikka K. Arffman, Laure Morin-Papunen, Stephen Franks, Marjo-Riitta Järvelin, Juha S. Tapanainen, Jouko Miettunen, Terhi T. Piltonen

**Affiliations:** 1 Department of Obstetrics and Gynecology, University Hospital of Oulu, University of Oulu, Medical Research Centre Oulu and PEDEGO Research Unit, Oulu, Kajaanintie 50, 90014 Oulu, Finland; 2 Institute of Reproductive and Developmental Biology, Imperial College, London, W12 ONN UK; 3 Department of Epidemiology and Biostatistics, Medical Research Council-PHE (Public Health England) Centre for Environment & Health, School of Public Health, Imperial College, London, W2 1PG UK; 4 Centre for Life Course Epidemiology, Faculty of Medicine, University of Oulu, FI-90014 Oulu, Finland; 5 Department of Obstetrics and Gynecology, University of Helsinki and Helsinki University Hospital, FI-00014 Helsinki, Finland; 6 Centre for Life Course Health Research, University of Oulu, Box 5000, FI-90014 Oulu, Finland; 7 Medical Research Centre Oulu, Oulu University Hospital and University of Oulu, Oulu, Finland

**Keywords:** Polycystic ovary syndrome, PCOS, Psychosis, Hirsutism, Testosterone

## Abstract

Polycystic ovary syndrome (PCOS) is a common endocrine disorder affecting up to 18% of women. Besides metabolic and fertility aspects, attention has lately been directed towards the detrimental effect of PCOS on psychological health. The objective of the study was to investigate whether women with PCOS are at higher risk for psychotic disorders. The study population derives from the Northern Finland Birth Cohort 1966 (*N* = 5889 women). The women with PCOS were identified by two simple questions on oligo-amenorrhea and hirsutism at age 31. Women reporting both symptoms were considered PCOS (*N* = 124) and asymptomatic women as controls (*N* = 2145). The diagnosis of psychosis was traced using multiple national registers up to the year 2016. Symptoms of psychopathology were identified using validated questionnaires at age 31. Women with PCOS showed an increased risk for any psychosis by age 50 (HR [95% CI] 2.99, [1.52–5.82]). Also, the risk for psychosis after age 31 was increased (HR 2.68 [1.21–5.92]). The results did not change after adjusting for parental history of psychosis, nor were they explained by body mass index or hyperandrogenism at adulthood. The scales of psychopathology differed between women with PCOS and non-PCOS controls showing more psychopathologies among the affected women. PCOS cases were found to be at a three-fold risk for psychosis, and they had increased psychopathological symptoms. PCOS should be taken into consideration when treating women in psychiatric care. More studies are required to further assess the relationship between PCOS and psychotic diseases.

**Supplementary Information:**

The online version contains supplementary material available at 10.1007/s00737-021-01195-4.

## Introduction

Polycystic ovary syndrome (PCOS) is the most common endocrine disorder of women, affecting up to 18% of reproductive-aged women (March et al. 2010). Unfortunately, this complex, chronic condition often remains undiagnosed and thus untreated (Piltonen et al. 2019; Teede et al. 2018). The diagnostic criteria of PCOS include ovulatory dysfunction/chronic anovulation, hyperandrogenism, and polycystic ovarian morphology; however, metabolic dysfunction and psychological illnesses are also dominant features of the syndrome.

Previous studies have shown that women with PCOS are at increased risk of psychological problems such as depression, anxiety, bipolar disorder, and disordered eating (Chen et al. 2020b, 2020c; Cooney et al. 2017; Karjula et al. 2017; Teede et al. 2018). The predisposing factors for these comorbidities remain unclear to date (Cooney et al. 2017; Teede et al. 2018). Even though genetic factors and family history of a psychiatric condition are the most common risk factors for psychological morbidities, various prenatal exposures and conditions during brain development, e.g., maternal obesity and stress, have also been shown to affect the probability of psychiatric morbidities later in life (Chen et al. 2020a; Kawai et al. 2004; Rice et al. 2007).

Psychosis and the specific diagnosis of schizophrenia represent the most severe forms of psychiatric disorders. Psychotic symptoms are characteristic of all psychotic syndromes, but the specific symptoms, duration, and effects on cognitive function vary (World Health Organization, 2018). Although several risk factors are known for psychosis, they explain only a minority of the cases. Indeed, several studies considering the etiology of psychosis show a multifactorial background with interaction between both genetic factors and the environment (Bearden & Forsyth, 2018; Matheson et al 2011). A family history of psychosis is considered the most important risk factor, but some prenatal and neonatal factors, such as obstetric complications or viral infections, also increase the risk (Matheson et al. 2011).

While psychiatric and psychological disturbances are strongly associated with PCOS, not much is known about more severe psychiatric diseases, schizophrenia, and other psychoses. Two studies have concluded that women with PCOS are at higher risk of schizophrenia (Cesta et al. 2016; Chen et al., 2020c); however, psychotic disorders other than schizophrenia were not investigated. Given that only a few studies have suggested elevated scores for psychotic symptoms (Borghi et al. 2017; Elsenbruch et al. 2003), further research is warranted.

Recently, there has been increasing interest in defining the roles of multiple hormonal factors and metabolic alterations in the development of psychosis, of which many are also associated with PCOS. It has been shown—although inconsistently—that hyperandrogenism could be a risk factor for psychosis in women (Misiak et al. 2018). A hypothesis regarding the protective action of estrogen has also been presented (Huber et al. 2004; Riecher-Rössler 2017), which could explain the sex differences in schizophrenia, i.e., later onset, less severe illness, and somewhat lower prevalence in women (Jongsma et al. 2019; Novick et al. 2016; Van Der Werf et al. 2014). Higher prolactin (PRL) levels in drug-naïve first-episode psychosis female patients have also been reported (Delgado-Alvarado 2019; Riecher-Rössler et al. 2013). As for metabolic traits, patients diagnosed with psychosis, even before antipsychotic medication, often display dyslipidemia (Misiak et al. 2017), higher waist circumference (Cordes et al. 2017), and glucose intolerance (Perry et al. 2019). Additionally, hypotheses suggesting an inflammatory origin for the pathogenesis of schizophrenia and other psychoses have been proposed (Upthegrove et al. 2014).

Recent studies have linked psychological disturbances in women with PCOS to the early development of the syndrome, possibly through in utero androgen exposure (Risal et al. 2019; Stener-Victorin et al. 2019). Moreover, as obesity and related insulin resistance and high serum insulin levels aggravate hyperandrogenism and cause fetal stress, they may also increase the risk of transgenerational transmission of the syndrome and induce psychiatric morbidity in the offspring (Cesta et al. 2020; Hatanaka et al. 2017; Kong et al. 2018; Stener-Victorin et al. 2019). Interestingly, emerging data imply that the traits of PCOS, including psychiatric morbidity, can be carried over several generations (Cesta et al. 2020; Risal et al. 2019; Stener-Victorin et al. 2019).

Against this background, the objective of the present work was to investigate whether women with PCOS are at higher risk for psychotic disorders. Here, for the first time, the psychosis risk was assessed in a population-based study-setting identifying women with PCOS symptoms and linking the data with the national datasets. We also used symptomatic psychopathology scales to assess susceptibility to psychosis (Miettunen et al. 2011; Miettunen & Jääskeläinen 2010). The study utilized the unique, longitudinal Northern Finland Birth Cohort dataset (NFBC1966), comprising all expected births in 1966 (*N* = 5889 women) with a longitudinal follow-up to age 50. We were also able to derive comprehensive national register data and a wide variety of possible confounding factors to investigate the risk of psychosis in affected women.

## Material and methods

### Study population

The NFBC1966 cohort population and validation of the characteristics related to PCOS diagnoses have been described previously (Karjula et al. 2017; Ollila et al. 2016; Taponen et al. 2003, 2004). In brief, the cohort includes individuals expected to be born in 1966 in Northern Finland (*n* total = 12,058; *n* female = 5889), altogether comprising 96.3% of total births in the region (Rantakallio 1988, University of Oulu 1966). This study utilized the cohort data collected at the age of 31 years as well as the national register data of psychiatric diagnoses and medications. The characteristics of the study groups are presented in Table 1. Data were gathered via a questionnaire sent to all cohort subjects and from clinical examinations.

**Table 1:** Clinical and socioeconomic characteristics of the control women and women with PCOS at age 31 in the Northern Finland Birth Cohort, 1966

Character	Controls	PCOS	*p* Value
	Q1 *n* = 2145	*n* = 124	
	Q2 *n* = 1376	*n* = 77	
BMI (kg/m^2^)	23.79 (4.32)	27.25 (6.86)	< 0.001
BMI			< 0.001
< 25 kg/m^2^	70.2 %	46.3 %	
≥ 25 kg/m^2^	21.6 %	28.1 %	
≥ 30 kg/m^2^	8.2 %	25.6 %	
Testosterone (nmol/l)	0.98 [0.75, 1.61]	1.39 [1.17, 1.79]	< 0.001
Testosterone (upper quartile)	21.8%	57.8%	< 0.001
FAI	2.11 [1.49, 2.97]	4.38 [2.75, 6.79]	< 0.001
FAI (upper quartile)	20.1%	67.1%	< 0.001
Parental history psychosis	6.2%	6.5%	0.921

Mean (standard deviation) or median with [25% lower quartile and 75% upper quartile] or proportions (%). The numbers of participants may vary due to lack of available data. *Q1* postal questionnaire, *Q2* clinical visit

**Fig. 1:**
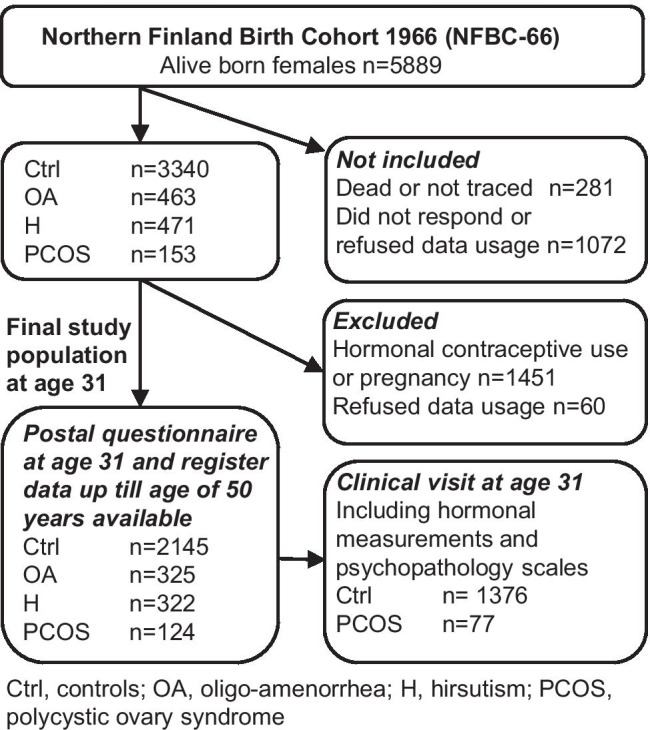
Flowchart of the study population

The questionnaire was sent to 5608 women of the cohort at age 31 (1996–1997), 4523 (81%) of whom responded. The questionnaire included questions concerning clinical factors such as weight and height. PCOS was screened with the following questions: (1) Is your menstrual cycle longer than 35 days more than twice a year? (considered as having oligo-amenorrhea, OA); and (2) Do you have excessive, bothersome body hair? (considered as having hirsutism, H). In total, 463 women reported OA, 471 reported H, and 153 reported having both symptoms (the latter category was considered to be women with PCOS). Women who were pregnant or using hormonal contraceptives or who did not permit the use of their personal register data were excluded from the final study population. The final analysis group at age 31 consisted of 2145 asymptomatic women (the control group), 325 women with isolated OA, 322 with isolated H, and 124 with PCOS. A flow chart of the study is presented in Fig. 1. Clinical examinations, including measurements of weight, height, serum testosterone (T), and free androgen index (FAI), were performed for 3127 women (76%).

All participants provided informed consent, and the study was approved by the Ethics Committee of the Northern Ostrobothnia District in Finland (EETTMK 94/2011).

### Identification of psychoses

For the identification of psychoses, several registers were utilized:

(1) Care Register for Health Care (CRHC) (1972–2016)

(2) Finnish outpatient registers: special health care (1998–2016) and primary health care (2011–2016)

(3) Social Insurance Institution (SII) registers: sick days (1974–1999), disability pensions (1974–2000), and reimbursable medicines (1974–2005)

(4) Finnish Centre for Pensions: disability pensions (1974–2016)

The identification of psychoses is described in more detail by Filatova et al. (2017). Diagnoses of non-organic psychosis were included up to 2016 (i.e., ICD‐8 295–299; ICD‐9 295, 2961E, 2962E, 2963E, 2964E, 2967, 297–299, 2988A, 2989X; ICD‐10 F20, F22‐F25, F28-F29, F302, F312, F315, F323, F333), representing the any psychosis group (Supplementary Table 1). For subgroup analysis, we separated the psychosis cases into schizophrenia and other psychosis as shown in Supplementary Table 1. For estimating the risk of psychosis between ages 31 and 50, we used the first onset of diagnoses of psychoses from age 31 onward.

### Parental psychosis

Parental psychosis was defined as a parent (mother and/or father) having non‐organic psychosis (i.e., ICD‐8 295–299; ICD‐9 295, 2961E, 2962E, 2963E, 2964E, 2967, 297–299; ICD‐10 F20, F22‐F29) at any time between 1964 and 2005. Information about parental psychosis was available from the disability pension register of the Finnish Centre for Pensions (1964–2005) and the CRHC (1972–2005), including outpatient registers from special health care (1998–2005). The proportions of parental psychosis in different study groups are shown in Table 1. There was no difference in the proportions of parental history of psychoses between the PCOS group (6.5%) and the controls (6.2%; *p* = 0.921).

### Psychopathology scales

As part of the clinical visit, the participants filled out a questionnaire consisting of mental health-related true/false (scored 0/1) questions collected from several psychological scales. The scales are used for the identification of psychopathological symptoms in individuals (Miettunen et al. 2011). The psychopathology scales used were the Social Anhedonia Scale (SAS), Physical Anhedonia Scale (PHAS), Perceptual Aberration Scale (PAS), Hypomanic Personality Scale (HPS), Bipolar II scale (BIP2), and Schizoidia Scale (SCHD). A more detailed description of the scales utilized in the same birth cohort has been published (Miettunen et al. 2011; Miettunen & Jääskeläinen 2010). In cases where more than 10% of the response items were missing, the scale was excluded.

### Confounding variables

#### Body mass index

In the clinical examinations, weight (kg) was measured on a digital scale which was calibrated regularly. Height (cm) was measured twice, using a standard and calibrated stadiometer. Body mass index (BMI) was calculated in kg/m^2^. If a measurement was missing, the self-reported values were used. No statistical difference was observed between the measured and the self-reported BMIs (Ollila et al. 2016).

### Testosterone and FAI

Serum T and the sex hormone binding globulin (SHBG) were assayed at age 31, as previously described (Karjula et al. 2017). Testosterone levels were assayed using Agilent triple quadrupole 6410 LC/MS equipment with an electrospray ionization source, operating in positive-ion mode (Agilent Technologies). At age 31, SHBG was assayed using fluoroimmunoassay (Wallac, Inc. Ltd., Turku, Finland). FAI was calculated as follows: 100xT (nmol/L)/SHBG (nmol/L).

#### Socioeconomic status/education

Socioeconomic status was based on years of education. The variable was classified into three groups according to the number of education years: ≤ 9, 9–12, and > 12 years.

### Statistical analysis

Statistical analyses were performed using SPSS version 25 for Windows. The analysis of categorical variables was carried out using the Chi-square test and Fisher’s exact test when appropriate. Cox regression analysis (using hazard ratios, HR) was used to estimate the association between PCOS and psychotic disorders. Kaplan-Meier survival analysis (Mantel-Cox estimation) was used to estimate the incidence of psychotic disorders. Parental history of psychosis, BMI, education, testosterone, or FAI was used as a covariate. The analyses of psychometric scales were done using Student’s *t*-test for variables with normal distributions and the Mann-Whitney *U*-test as a non-parametric test. The data was also analyzed after removing women with a diagnosis of psychosis before age 31 to assess the incidence after the establishment of PCOS symptoms. The results are reported as means with standard deviations (SD), medians (25% and 75% quartiles), prevalence (%), and HR with a 95% confidence interval (CI). *p* Values < 0.05 were considered statistically significant.

## Results

### Lifetime prevalence and incidence of psychosis

**Fig. 2:**
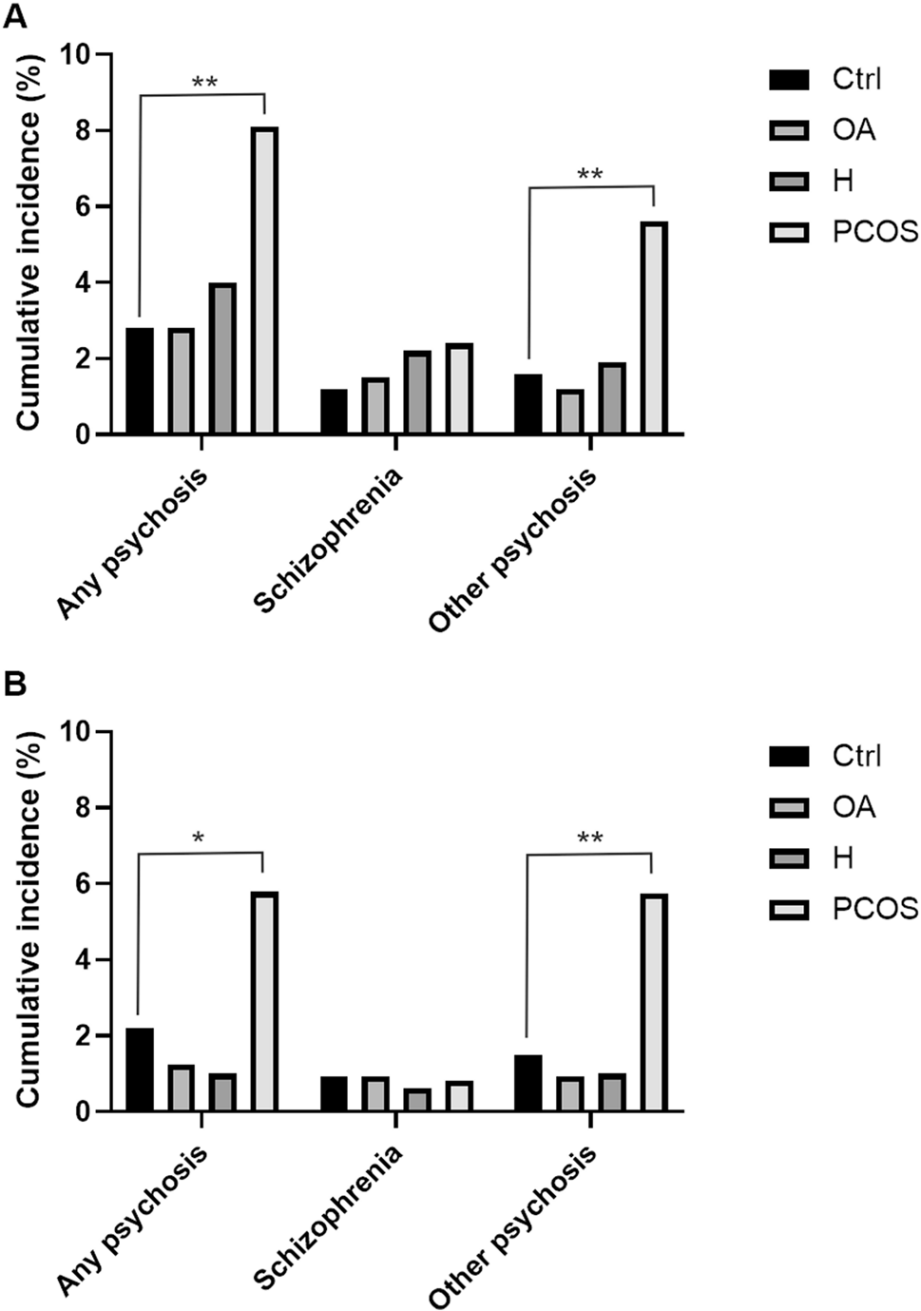
Cumulative incidence of psychosis **a** until age 50 and **b** from age 31 to 50 years, in women with isolated oligo-amenorrhea (OA), women with isolated hirsutism (H), and women with PCOS (OA+H) compared to controls (Ctrl) (*p < 0.05, **p < 0.01, ***p < 0.001)

The lifetime prevalence (until age 50) of any psychoses in women with PCOS was 8.1% (compared to 2.8% for controls; *p* = 0.004). The prevalence of schizophrenia was not significantly higher in the PCOS group (2.4% vs. 1.2%; *p* = 0.245), but the number of cases was severely limited. However, the lifetime prevalence for psychoses other than schizophrenia was increased in PCOS: 5.6% vs. 1.6% (*p* = 0.006) (Fig. 2A).

When considering the diagnoses of psychosis from age 31 to 50, the incidence of any psychosis in PCOS women was 5.8%, compared to 2.2% for controls (*p* =0.023). There were no differences in diagnoses of schizophrenia between the PCOS group (0.8%) and controls (0.9%; *p* =0.96) during this period, whereas the incidence of other psychoses was 5.7% (compared to 1.5% for controls; *p* =0.004) (Fig. 2B). No differences were found between women with isolated OA or H compared to controls; thus, these two groups were not included in further analyses (Fig. 2A,B).

### The risks of lifetime diagnoses of psychosis until age 50

**Fig. 3:**
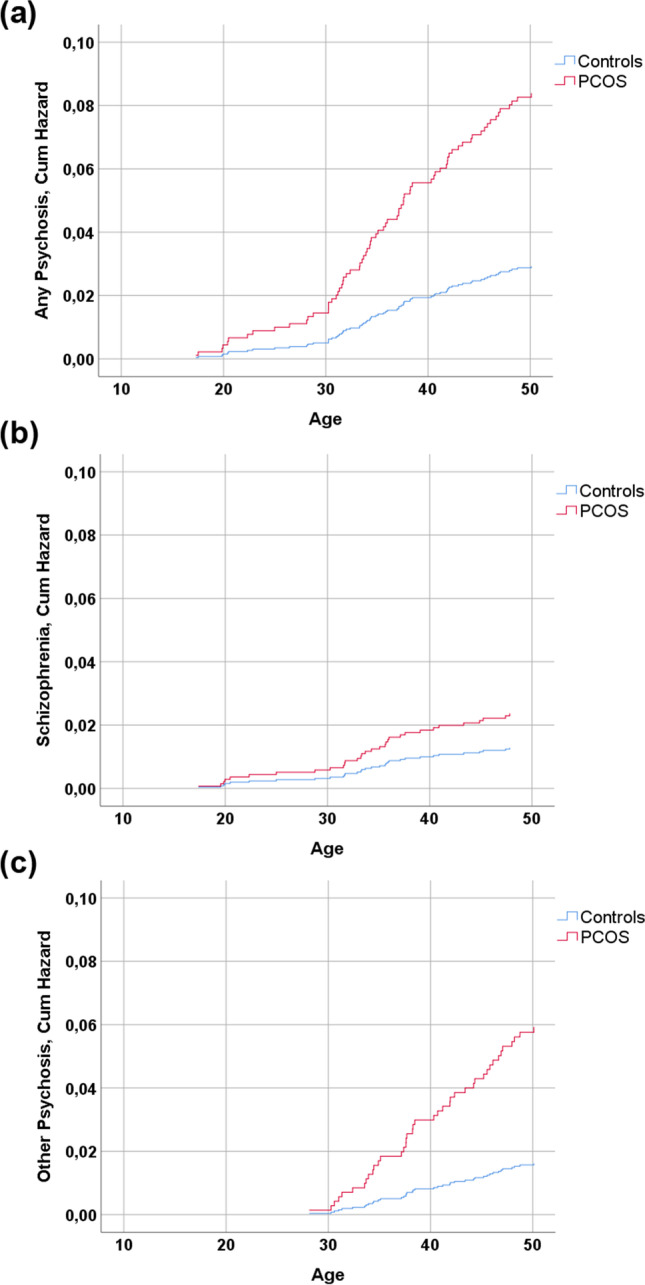
Cumulative hazard functions of age at onset of psychosis in women with PCOS and non-PCOS controls: **a** any psychosis, **b** schizophrenia, and **c** other psychosis up to age 50. The figures also include hazard ratios (HR) and their 95% confidence intervals from Cox regression analysis for **a** psychoses, **b** schizophrenia, and **c** other psychosis. The data has been adjusted for parental history of psychosis. On the Y axis, the incidence is presented as part of the whole, where 1.0 represents the whole study population

In the Cox regression model (Fig. 3), the risk of any psychosis until age 50 in women with PCOS symptoms was increased (HR 2.99, 95% confidence interval [95% CI] 1.53–5.83). After adjusting for parental psychoses, the risk remained high (HR 2.98, 95% CI 1.52–5.82). The risk of schizophrenia did not reach statistical significance (HR 2.04, 95% CI 0.62–6.74), likely due to the low number of cases in the PCOS group. In contrast, the risk of other psychoses was higher in women with PCOS (HR 3.66, 95% CI 1.62–8.26), and adjusting for parental history of psychosis did not affect the result (HR 3.62, 95% CI1.61–6.10) even though it was still strongly associated with the risk of all psychoses (data not shown).

### The risks of psychoses from age 31 to age 50

The Cox regression analysis showed that the risk of having any psychoses from age 31 to age 50 was elevated significantly in women with PCOS (unadjusted HR 2.69, 95% CI 1.21–5.94) and this result did not change even after adjusting for parental history of psychoses (adj. HR 2.68, 95% CI 1.21–5.92). Furthermore, after adjusting for BMI and education, the risks were not reduced (Table 2).

**Table 2:** The risk of any psychoses, schizophrenia, and other psychosis in women with PCOS between ages 31 and 50 in the Cox regression model. The risk is presented in the form of hazard ratios

	Any psychosis	Schizophrenia	Other psychosis
PCOS *n* = 121–122* Ctrl *n* = 2132–2139*	*n* = 7 (5.8%) *n* = 47 (2.3%)	*n* = 1 (0.8%) *n* = 20 (0.9%)	*n* = 7 (5.7%) *n* = 32 (1.5%)
Unadjusted (HR [95% CI])	2.69 (1.21–5.94)	0.89 (0.12–6.62)	3.95 (1.74–8.95)
Adj. BMI at age 31	2.81 (1.24–6.37)	0.89 (0.12–6.78)	4.16 (1.78–9.74)
Adj. parental history of psychosis	2.68 (1.21–5.92)	0.89 (0.12–6.59)	3.95 (1.74–8.94)
Adj. education	2.64 (1.19–5.85)	0.87 (0.12–6.46)	3.87 (1.71–8.80)

*HR* hazard ratio, *CI* confidence interval, *BMI* body mass index. *Numbers vary due to missing data from some individuals for specific variables

### The effect of hyperandrogenism on the risk of psychoses

Of the women with PCOS who attended the clinical examination and blood tests at the age of 31, 9.5% received new diagnoses of psychosis between ages 31 and 50, compared to 2.8% for the controls (unadjusted HR 3.58, 95% CI 1.60–8.04). When adjusting for testosterone (at age 31), the risk was slightly reduced (HR 3.03, 95% CI 1.26–7.31), as was the case also when adjusting for FAI (HR 2.85, 95% CI 1.09–7.49) (Table 3). However, neither serum testosterone nor FAI was independently associated with the risk of psychosis (data not shown).

**Table 3:** The risk of any psychosis, schizophrenia, and other psychosis in women with PCOS between ages 31 and 50 in a Cox regression model adjusted with BMI, testosterone, and free androgen index (FAI). The data includes only women attending clinical examination at age 31 and for whom testosterone and sex hormone binding globulin (SHBG) measurements are thus available

	Any psychosis	Schizophrenia	Other psychosis
PCOS *n* = 74–75* Ctrl *n* = 1368–1372*	*n* = 7 (9.5%) *n* = 37 (2.8%)	*n* = 1 (1.3%) *n* = 14 (1.0%)	*n* = 7 (9.3%) *n* = 26 (1.9%)
Unadjusted [HR (95%CI)]	3.58 (1.60–8.04)	1.32 (0.17–10.06)	5.08 (2.21–11.70)
Adj. BMI at age 31	3.94 (1.72–9.04)	1.53 (0.20–11.89)	5.34 (2.25–12.68)
Adj. testosterone	3.03 (1.26–7.31)	0.98 (0.12–8.29)	4.53 (1.82–11.32)
Adj. FAI	2.85 (1.09–7.49)	1.52 (0.17–13.72)	3.87 (1.41–10.59)

*HR* hazard ratio, *CI* confidence interval, *BMI* body mass index, *FAI* free androgen index. *Numbers vary due to missing data from some individuals for specific variables

### The results of psychopathology scales

For women with PCOS symptoms, the scales for PAS were significantly higher than for controls (*p* = 0.024). The mean (SD) HPS score for the PCOS group was 14.88 (8.59), compared to 11.61 (6.97) in controls (*p* = 0.002), and for the SCHD, it was 3.14 (1.49) in PCOS vs. 2.77 (1.38) in controls. No statistically significant differences were found between PCOS cases and controls in SAS, PHAS, or BIP2 (Table 4).

**Table 4:** Psychopathology scales in control women and in women with PCOS at age 31 without previous diagnosis of psychosis

Scale	Controls *n* = 1277–1281* median [IQR] or mean (SD)	PCOS *n* = 71–72* median [IQR] or mean (SD)	*p* Value
SAS	7.0 [5.0, 10.0]	8.0 [6.0, 11.0]	0.065
PHAS	12.00 [9.0, 16.0]	13.0 [9.0, 16.0]	0.330
PAS	1.0 [0, 3.0]	2.0 [1.0, 4.0]	0.024
HPS	11.61 (6.97)	14.88 (8.59)	0.002
BIP2	10.32 (3.68)	11.29 (4.77)	0.093
SCHD	2.77 (1.38)	3.14 (1.49)	0.024

The results are reported as mean (standard deviation) or median with [25% lower quartile and 75% upper quartile] when appropriate. The differences are analyzed using Student’s *t*-test or Mann-Whitney *U*-test. The psychopathology scales used were the Social Anhedonia Scale (SAS), Physical Anhedonia Scale (PHAS), Perceptual Aberration Scale (PAS), Hypomanic Personality Scale (HPS), Bipolar II scale (BIP2), and Schizoidia Scale (SCHD). *Numbers vary due to missing data from some individuals for specific variables

## Discussion

This community-based population study shows for the first time that women with PCOS are at a three-fold increased risk of psychoses compared to non-PCOS controls, at least until age 50. Indeed, the fact that the lifetime prevalence of psychosis was 8.1% in PCOS subjects compared to 2.8% in controls warrants attention. An elevated risk of psychoses was also detected during the follow-up period started after the establishment of the PCOS diagnosis (ages 31–50). The results were independent of parental history of psychosis, the most strongly predisposing factor for severe mental illness (Matheson et al. 2011). The results also indicated that isolated PCOS symptoms (H or OA alone), BMI, and biochemical hyperandrogenism in adulthood were not major contributors to psychosis risk in women with PCOS.

Recently, there has been increasing interest in psychological distress in women with PCOS. Affected women have been found to have an increased risk of anxiety, depression, bipolar disorder, and eating disorders compared to non-PCOS controls (Cesta et al. 2016; Chen et al., 2020b, 2020c; Cooney et al. 2017; Karjula et al. 2017; Teede et al. 2018). However, previous evidence for the risk of severe mental disorders in affected women is limited, highlighting the need to explore this area. Interestingly, a previous study showed that women at their first admissions due to schizophrenia more often showed PCOS-related symptoms (such as pre-existing menstrual disturbances, hair loss, hirsutism, and infertility) than age-matched healthy women (Riecher-Rössler 2002). A previous study by Cesta et al., also performed on a Nordic population, did not assess the risk of other psychoses in PCOS patients; however, they did report an increased risk of schizophrenia, in contrast to our results (Cesta et al. 2016). In addition, a recent Taiwanese cohort study reported a greater than six-fold risk of schizophrenia in PCOS subjects (Chen et al. 2020c). For our study, the number of PCOS cases with schizophrenia was likely resulting in lack of power for analysis, and thus no reliable conclusions can be drawn regarding the association of schizophrenia and PCOS. However, in line with our data, another Taiwanese population-based cohort study found the HR for schizophrenia to be nonsignificant during a 10-year follow-up period (HR 0.84, 95% CI 0.43–1.59) (Hung et al. 2014).

The results of the psychopathology scale analysis, including PAS, HPS, and SCHD, indicated higher psychopathology in women with PCOS. These psychopathology scales are known to be associated with psychotic diseases and/or symptoms (Miettunen et al. 2011), thus supporting the notion of increased risk of psychosis in women with PCOS. Previously, two studies investigated psychological disturbances using the symptom checklist-90 (SCL-90) scales and reported increased psychoticism scores in women with PCOS (Borghi et al. 2017; Elsenbruch et al. 2003), in line with our results.

With increasing evidence of psychological distress in PCOS, the number of studies on the mechanisms behind the morbidity is also increasing. These studies consider the factors associated with the syndrome, such as hyperandrogenism, obesity, and insulin resistance (Cooney et al. 2017; Greenwood et al. 2019). Other recent studies considering the predisposing factors for psychotic disorders and other psychiatric diseases have focused on hormonal and metabolic factors (Misiak et al., 2017, 2018; Perry et al., 2019).

It has been reported that patients with severe mental disorders are more commonly obese, possibly due to antipsychotic medication as well as an unhealthy lifestyle (De Hert et al., 2011). In the present study, the risk of psychosis did not decrease after adjusting for BMI at age 31. In line with our finding, higher BMI was poorly correlated to psychosis risk in a previous study (Shah et al. 2019). Considering psychological distress in PCOS subjects, the syndrome itself has been shown to be an independent risk factor, although a high BMI might serve as a predisposing factor for depression and anxiety in the affected women (Cooney et al. 2017; Greenwood et al. 2019). The fact that weight gain aggravates PCOS symptoms while also increasing its prevalence supports the link between PCOS and psychological distress (Teede et al. 2013, 2018). Insulin resistance also appears to play a role in the psychological morbidity in PCOS, and in support of this, metformin therapy has been shown to reduce risk for bipolar disorder in this population (Chen 2020a).

Previous studies have implied a protective role for estrogen regarding psychosis risk (Huber et al.^2004^; Anita Riecher-Rössler 2017), while higher and rising estradiol levels during the menstrual cycle have been shown to relieve psychotic episodes and symptoms (Bergemann et al. 2007). Furthermore, physiological hypoestrogenism related to menopause also seems to be associated with an increased risk of schizophrenia (Searles et al. 2018). Theoretically, the link between low estrogen levels and psychosis risk may also relate to anovulation and a lack of high estrogen peaks in PCOS subjects due to absent menstrual cycles.

In the present study, the risk of psychosis in women with PCOS was slightly decreased after adjusting for T or FAI; however, in the whole population, high serum androgen levels in adulthood did not present as an independent risk factor (data not shown). Moreover, isolated PCOS symptoms (H or OA) were not associated with the risk of psychosis. This could underline the specific role of early exposure to hyperandrogenism in women with PCOS, as systemic hyperandrogenism in adulthood does not seem to present as a high-risk factor for psychosis in the general female population (Misiak et al., 2018). Indeed, exposure to hyperandrogenism in utero appears to provoke mental disturbances, in addition to the PCOS phenotype, in animal models (Stener-Victorin et al., 2019). On the other hand, high testosterone levels in the perinatal period promote abnormal synaptic development and sexual dimorphism in the brain, providing mechanisms for an increased risk of neurocognitive impairments such as ADHD and autism (Auyeung et al. 2013; Hatanaka et al. 2017). This could also provide the link between PCOS and psychiatric comorbidity related to the syndrome.

Given that family history is the most important factor for psychosis risk, transgenerational transmission (TT) should also be considered. In PCOS, TT and related hyperandrogenism are evidenced by recent studies following the offspring of PCOS-like mice to the third generation, supported by human epidemiological data (Mimouni et al. 2021; Risal et al. 2019). As for inherited traits of psychological morbidity, a recent study of register data showed that the daughters of mothers with PCOS presented more often with male-prominent neuropsychiatric diseases—i.e., autism spectrum disorders and ADHD (Cesta et al., 2020). Whether this also applies to more severe mental illnesses, such as psychosis, remains to be investigated.

There are strengths but also some limitations in the present study. The strengths include the population-based, longitudinal approach. Moreover, this prospectively collected data was also linked to the comprehensive national register data regarding psychiatric diagnoses. The Finnish national registers have been shown to be good and reliable data sources (Perälä et al., 2007). An extensive list of confounding factors was also available, as well as the history of parental psychosis. The fact that we were able to identify a female population with a high risk of developing psychiatric disease using only two simple questions about menstrual cycles and excessive hair growth is worth noting. The self-reported diagnosis of PCOS might still be considered a limitation, even though a hormonal, metabolic, and psychological profile typical for women with PCOS has been described extensively in our previous studies with this population (Karjula et al. 2017; Ollila et al. 2016; Taponen et al. 2003, 2004). Given that psychoses are highly health-burdening conditions, the related possible low attendance to cohort data collection could affect the result as the PCOS diagnosis would not have been established for these individuals (Haapea et al., 2008). Additionally, as existing psychosis and antipsychotic medication may cause excessive weight gain and menstrual irregularities, thus increasing the probability of PCOS symptoms, we conducted an analysis of psychosis incidence after the establishment of PCOS diagnosis at the age of 31 (De Hert et al., 2011; Gleeson et al. 2016; Teede et al. 2013, 2018). However, this age point may be relatively late to assess schizophrenia as the disease is commonly diagnosed in one’s twenties (Van Der Werf et al. 2014). One of the major limitations was the low number of schizophrenia and psychosis cases among the PCOS population. For schizophrenia, the high confidential interval for risk implies that there was not enough power for the analysis to detect the difference in the risk. Given all mentioned before, this may have led to underestimation of the prevalence of severe psychiatric comorbidity in women with PCOS. This underlines the need for further studies with bigger study populations. Furthermore, the current population-based sample was unselected, but genetically homogeneous and the sample consisted mainly of women with white Caucasian background. Based on this, the results may not be generalizable to more heterogeneous populations.

## Conclusions

The fact that women with PCOS seem to have a high risk of psychiatric comorbidities warrants devoting more resources and greater efforts to improve awareness, treatment modalities, and quality of life (Piltonen et al. 2019; Teede et al. 2018). Especially as the physical health of patients with severe mental illnesses has been shown to be poorly examined and treated (De Hert et al. 2011; Lambert et al. 2018), guidelines and recommendations for treating somatic diseases in women with severe mental illnesses are needed.

Based on the present results, the association of severe mental disorders and PCOS should be acknowledged in clinical practice. Future research should focus on investigating the mechanisms behind this association, thus enabling prevention and new treatment strategies.

## Supplementary Information

Below is the link to the electronic supplementary material.

Supplementary file1 (DOCX 19 KB)

## Data Availability

NFBC data is available from the University of Oulu, Infrastructure for Population Studies. Permission to use the data can be applied for research purposes via electronic material request portal. In the use of data, we follow the EU general data protection regulation (679/2016) and Finnish Data Protection Act. The use of personal data is based on cohort participant’s written informed consent at his/her latest follow-up study, which may cause limitations to its use. Please, contact NFBC project center (NFBCprojectcenter@oulu.fi) and visit the cohort website (www.oulu.fi/nfbc) for more information.
