# Supplementary material for: A population-based follow-up study shows high psychosis risk in women with PCOS
Source: Arch Womens Ment Health. 2021 Nov 29;25(2):301–11. doi: 10.1007/s00737-021-01195-4 (PMC8921102; doi:10.1007/s00737-021-01195-4)
Supplement: Supplementary file 1 — Supplementary file1 (DOCX 19 KB) [file 737_2021_1195_MOESM1_ESM.docx]

**Supplementary Table 2. Psychotic disorder categories used in the study. The diagnoses with light grey background represent the schizophrenia group and those with dark grey the other psychosis group. Any psychosis group includes all the diagnoses.**

| **Diagnosis** |  | **ICD-8 code (1968-1986)** |  | **ICD-9 code (1987-1995)** |  | **ICD-10 code (1996-)** |
| --- | --- | --- | --- | --- | --- | --- |
|  |  |  |  |  |  |  |
| **Schizophrenia** |  | 295, 2954 |  | 295, 2954 |  | F20 |
| **Schizophrenia spectrum** |  |  |  |  |  |  |
| **Schizoaffective disorder** |  | 2957 |  | 2957 |  | F25 |
| **Delusional disorder** |  | 297 |  | 297 |  | F22 |
|  |  |  |  |  |  |  |
| **Bipolar disorder (with psychotic features)** |  | 1961-2969 |  | 2962E, 2963E, 2964E, 2967 |  | F302, F312, F315 |
| **Major depressive disorder (with psychotic features)** |  | 2960, 2980 |  | 2961E |  | F323, F333 |
| **Brief psychosis** |  | 298 (excluding 2980) |  | 2988 |  | F23, F24 |
| **Other non-organic psychoses** |  | 299 |  | 2989 |  | F28, F29 |
|  |  |  |  |  |  |  |

ICD, International classification of diseases.
